# Network of Mediators for Vascular Inflammation and Leakage Is Dysbalanced during Cytoreductive Surgery for Late-Stage Ovarian Cancer

**DOI:** 10.1155/2019/5263717

**Published:** 2019-07-17

**Authors:** Sven Klaschik, Jennifer Gehlen, Claudia Neumann, Mignon-Denise Keyver-Paik, Martin Soehle, Stilla Frede, Markus Velten, Andreas Hoeft, Tobias Hilbert

**Affiliations:** ^1^Department of Anesthesiology and Intensive Care Medicine, University Hospital Bonn, Venusberg-Campus 1, 53127 Bonn, Germany; ^2^Department of Gynecology and Obstetrics, University Hospital Bonn, Venusberg-Campus 1, 53127 Bonn, Germany

## Abstract

**Background:**

Cytoreductive surgery (CS) in late-stage ovarian cancer patients is often challenging due to extensive volume shifts, and high fluid intake may provoke postoperative complications. Expression of vasoactive mediators is altered in cancer patients, which may affect systemic vascular function. We sought to assess how serum levels of vasoactive markers and mediators change during CS in ovarian cancer.

**Methods:**

Following IRB approval and informed consent, pre- and postoperative serum samples were analyzed in 26 late-stage ovarian cancer patients using multiplex protein arrays and ELISA.

**Results:**

The proinflammatory cytokines and chemokines IL-6, IL-8, and CCL2 were significantly elevated after 24 hrs compared to the baseline values, with IL-6 and IL-8 being most prominently increased. While ANGPT1 remained unchanged after surgery, its competitive antagonist ANGPT2 was significantly increased. In contrast, serum levels of the ANGPT receptor TIE2 were decreased to 0.6 of the baseline values. While VEGF-D, E-selectin, P-selectin, ICAM-1, and PECAM-1 remained unchanged, serum activity of both thrombomodulin and syndecan-1 was significantly increased following surgery.

**Conclusion:**

We identified a regulatory network of acute-phase reaction during CS in late-stage ovarian cancer. This suggests that IL-6 exerts positive regulation of other proinflammatory mediators and, by upregulating ANGPT2 and suppressing ANGPT1, induces a serum profile that promotes vascular leakage. This may contribute to the observed hemodynamic alterations during CS procedures.

## 1. Introduction

In recent years, improved outcome and prolonged survival has been demonstrated for cytoreductive surgery (CS) in ovarian cancer (OC) patients even in advanced disease stages [1]. These procedures are, from the anesthesiologist's point of view, often challenging due to severe intra- and perioperative volume shifts. Despite advanced monitoring including transpulmonary thermodilution and pulse contour analysis usually been performed, intraoperative hemodynamic stabilization often requires extensive fluid intake as well as positive fluid balance [2]. This may provoke postoperative anesthesiological and surgical complications such as respiratory failure, impaired wound healing, and surgical site infections [2, 3]. Albeit still remaining elusive, the pathogenetic background for the observed need for extensive fluid resuscitation in this patient population is likely to be multicausal.

Cancer tissues express a number of peptides that indicate and promote tumor progression, such as proteases, growth factors, or proinflammatory cytokines [4, 5]. Since these soluble proteins can be detected in peripheral blood, their altered expression levels relative to healthy individuals can be used to estimate tumor load and patients' prognosis. Some of these markers possess vasoactive properties, thus altering vascular function not only in the tumor microenvironment but also in systemic perfusion [6]. It has been demonstrated that surgery for OC alters postoperative systemic expression of inflammatory mediators and that these relatively short-term changes in serum levels can help predict infective complications [7]. Furthermore, alterations in point-of-care coagulation tests as well as danger-associated molecular patterns (DAMPs) during the course of surgery suggest dynamic behavior of functional serum proteins and circulating molecules [8, 9]. However, short-term changes in serum activity during CS of mediators that may induce or at least promote hemodynamic destabilization through vascular inflammation and increased permeability [10] have not been described yet. It was our aim to assess how serum levels of selected vasoactive markers and mediators are changed during CS in OC patients. These peptides were measured preoperatively as well as in the early postoperative period.

## 2. Materials and Methods

### 2.1. Study Design and Patient Information

This observational study was conducted in accordance with the Declaration of Helsinki and after the approval by the institutional review board (IRB) of the University of Bonn (protocol number 360/16, date of approval March 21, 2018). Patients being scheduled for laparotomy to perform CS due to late-stage OC and who gave written informed consent were prospectively screened to participate in the study. Exclusion criteria were as follows: patient age < 18 years, refusal or inability to provide written informed consent, and pregnancy. Prior to anesthesia induction, a thoracic epidural catheter was usually placed for postoperative analgesia. All patients received anesthesia induction according to standard procedures including intubation, femoral arterial line, central venous catheterization, and urinary catheter. Anesthesia was induced with sufentanil, propofol, and rocuronium and was maintained with either isoflurane or sevoflurane and by continuous infusion of remifentanil. In addition to the standard monitoring, advanced hemodynamic monitoring was performed using the VolumeView system (EV-1000, Edwards Lifesciences Corp., Irvine, CA, USA). Intraoperative management followed a goal-directed algorithm. Crystalloid fluids, norepinephrine, and dobutamine were administered to keep cardiac index > 3.0 l/min∗m^2^, stroke volume variation < 20%, and mean arterial pressure (MAP) > 65 mmHg. Red blood cell concentrates and fresh frozen plasma were substituted according to the recent transfusion guidelines. The responsible anesthetist was not part of the study team. Depending on the progress of cancer, some patients were treated with hyperthermic intraperitoneal chemotherapy. Upon completion of the surgical procedure, anesthesia was terminated, and patients were extubated if a stable respiratory situation was provided. Subsequently, patients were transferred to the ICU for postoperative care.

### 2.2. Assessment of Mediators in Patient Serum

Ten mls of blood was drawn before the beginning of surgery (baseline) as well as 24 hrs following anesthesia induction. Coagulated samples were centrifuged (3,000 rpm, 4°C, 10 min), and serum aliquots were stored at -80°C° for subsequent analysis.

Samples were analyzed for the following peptides: interleukin (IL)-6, IL-8, CC chemokine ligand 2 (CCL2), angiopoietin 1 (ANGPT1), ANGPT2, tyrosine kinase with Ig and EGF homology domains 2 (TIE2), vascular endothelial growth factor isoform D (VEGF-D), E-selectin, P-selectin, intercellular adhesion molecule 1 (ICAM-1), platelet endothelial cell adhesion molecule (PECAM-1), thrombomodulin (THBD), and syndecan-1 (SDC1).

All peptides except for ANGPT1 and IL-8 were detected using custom-made Luminex™ multiplex arrays purchased from R&D Systems (Minneapolis, MN, USA) according to the manufacturer's protocol. Arrays were analyzed on a MAGPIX™ reader (Luminex Corp., Austin, TX, USA). ANGPT1 and IL-6 were measured using commercially available ELISA kits (R&D Systems) according to the manufacturer's instructions. Results are given in pg/ml serum. All experimental analyses were performed in duplicates. Out of these results, the mean value was calculated and used for further statistical analyses. All personnel performing the serum analyses were blinded for the intra- and postoperative patient data.

### 2.3. Statistical and Bioinformatical Analysis

Data were transferred into MS Excel (Microsoft Corp., Redmond, CA, USA). Statistical analysis and visualization was performed using GraphPad PRISM 5 (La Jolla, CA, USA). Unless otherwise stated, data are presented as median values with 25^th^ and 75^th^ percentiles. Significance of differences was tested using the Wilcoxon signed-rank test, and *p* values <0.05 were considered statistically significant.

Further evaluation of the data was performed using Ingenuity Pathway Analysis (IPA; Ingenuity Systems Inc., Redwood City, CA, USA). IPA maps each gene within a global molecular network derived from information contained in the Ingenuity Pathway Knowledge Base. A *network* in IPA is defined as a graphical representation of the molecular relationships among specific genes, represented as nodes, and the biological relationship between nodes shown as a connecting line. All connections are supported by published data from peer review and online available articles stored in the Ingenuity Pathway Knowledge Base and/or in the PubMed database. IPA ranks all genes based on their connectivity, using a generalization of the concept of node degree, which measures the number of single genes to which a gene is connected (for further information, see https://analysis.ingenuity.com/pa/info/help/Ingenuity_Network_Algorithm_Whitepaper_FINAL(2).pdf, and for further details, please refer to Calvano et al. [11]). Results from the IPA analysis were furthermore consolidated by a comprehensive literature search on each interaction.

The datasets generated and analyzed during the current study are available from the corresponding author on reasonable request.

## 3. Results

A total of 30 patients were recruited to participate in the study. Diagnosis of ovarian cancer was confirmed by histopathological analysis in all but 4 patients, which were excluded afterwards. Median age was 70 (57-75) years. The median duration of surgery was 456 (323-542) min, while the duration of anesthesia was 618 (506-689) min.  Table 1 gives an overview of the basic patients' characteristics and the procedural details.

### 3.1. Cytokines and Chemokines

**Figure 1:**
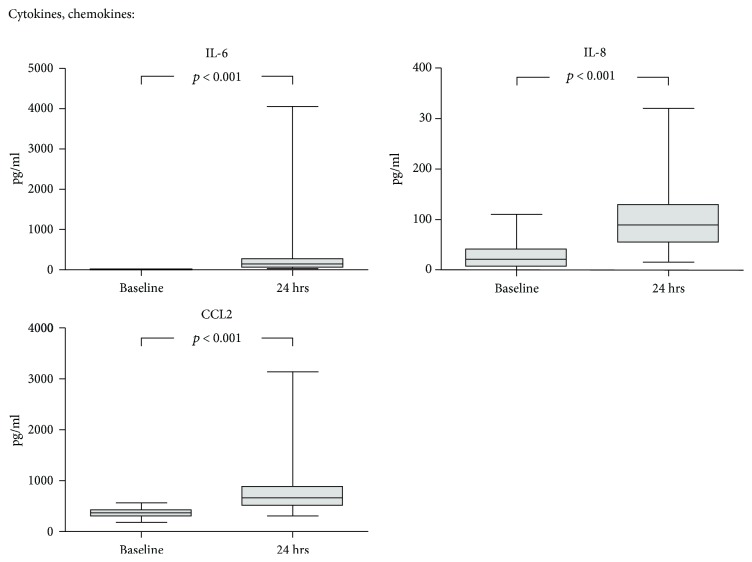
Dynamics of cytokines and chemokines during CS. Serum was collected prior to cytoreductive surgery (CS) as well as after 24 hrs, and a panel of cytokines and chemokines was measured by multiplex analysis and ELISA. Median with 25^th^ and 75^th^ percentiles (boxes) and minimum and maximum (whiskers), *n* = 26, Wilcoxon signed-rank test.

Serum was collected prior to surgery as well as after 24 hrs, and a panel of vascular activation and damage markers and mediators was measured by multiplex analysis and ELISA. The proinflammatory cytokines and chemokines IL-6, IL-8, and CCL2 were all significantly increased after 24 hrs compared to baseline values (Figure 1). Among all measured serum proteins, IL-6 and IL-8 were most prominently increased postoperatively with a median fold change of 10.4 (7.2-47.9) and 3.7 (1.2-9.7), respectively. However, both showed a wide interindividual variation.

### 3.2. Leakage-Related Mediators

**Figure 2:**
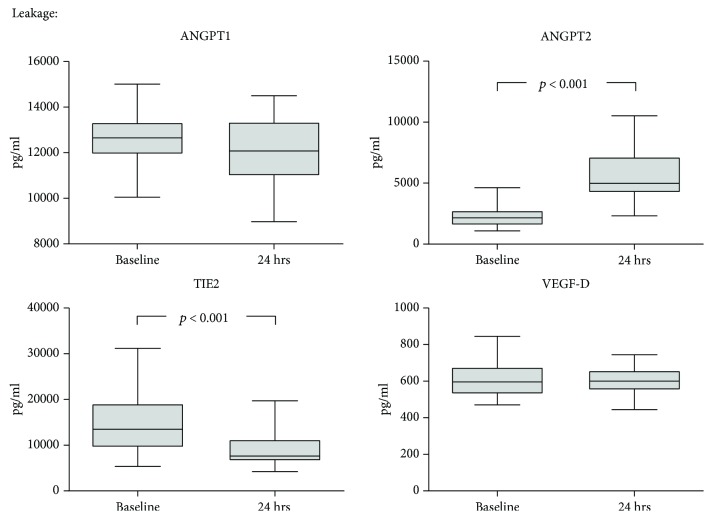
Dynamics of leakage-related mediators during CS. Serum was collected prior to cytoreductive surgery (CS) as well as after 24 hrs, and a panel of mediators of endothelial leakage was measured by multiplex analysis and ELISA. Median with 25^th^ and 75^th^ percentiles (boxes) and minimum and maximum (whiskers), *n* = 26, Wilcoxon signed-rank test.

Figure 2 gives the kinetics of the leakage-affecting mediators ANGPT1 and ANGPT2 the soluble form of their common receptor TIE2 and of VEGF isoform D. While ANGPT1 remained unchanged after surgery, its competitive antagonist ANGPT2 was significantly increased (median fold change 2.6 (2.1-2.9). Serum levels of TIE2 were likewise altered, with a median decrease to 0.6 (0.5-0.9) of the respective baseline values. VEGF-D remained unchanged after surgery.

### 3.3. Adhesion Molecules

**Figure 3:**
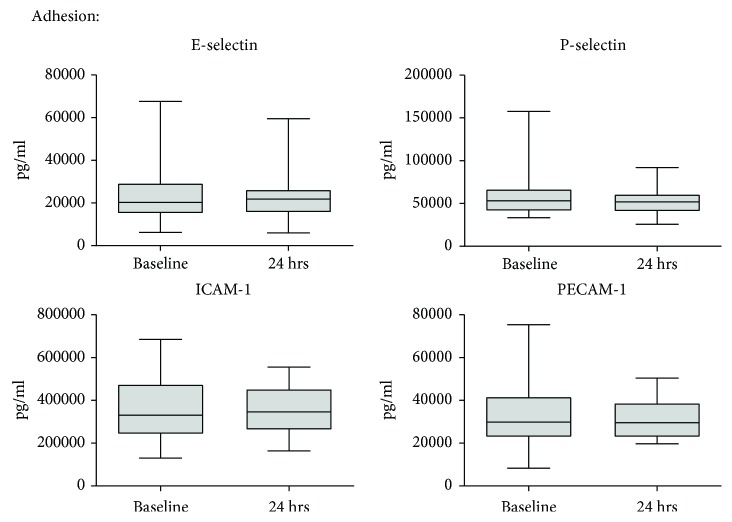
Dynamics of endothelial adhesion molecules during CS. Serum was collected prior to cytoreductive surgery (CS) as well as after 24 hrs, and a panel of endothelial adhesion molecules was measured by multiplex analysis. Median with 25^th^ and 75^th^ percentiles (boxes) and minimum and maximum (whiskers), *n* = 26, Wilcoxon signed-rank test.

Soluble forms of endothelial surface adhesion molecules were detectable in the patients' serum at baseline (Figure 3). However, the levels of E-selectin, P-selectin, ICAM-1, and PECAM-1 were not changed during the course of surgery.

### 3.4. Markers Related to Coagulation

**Figure 4:**
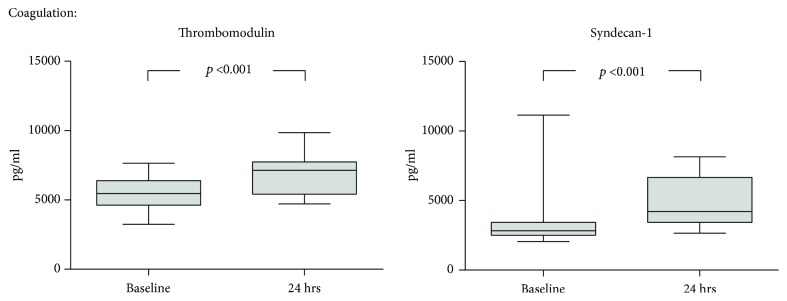
Dynamics of markers of blood coagulation during CS. Serum was collected prior to cytoreductive surgery (CS) as well as after 24 hrs, and a panel of peptides modulating local blood coagulation was measured by multiplex analysis. Median with 25^th^ and 75^th^ percentiles (boxes) and minimum and maximum (whiskers), *n* = 26, Wilcoxon signed-rank test.

THBD and SDC1 are both endothelial membrane proteins with implications for local coagulation and inflammation. Their serum levels increased during surgery compared to baseline conditions, with a median 1.3 (1.2-1.5) fold change for THBD and 1.5 (1.1-2.2) for SDC1 (Figure 4).

**Figure 5:**
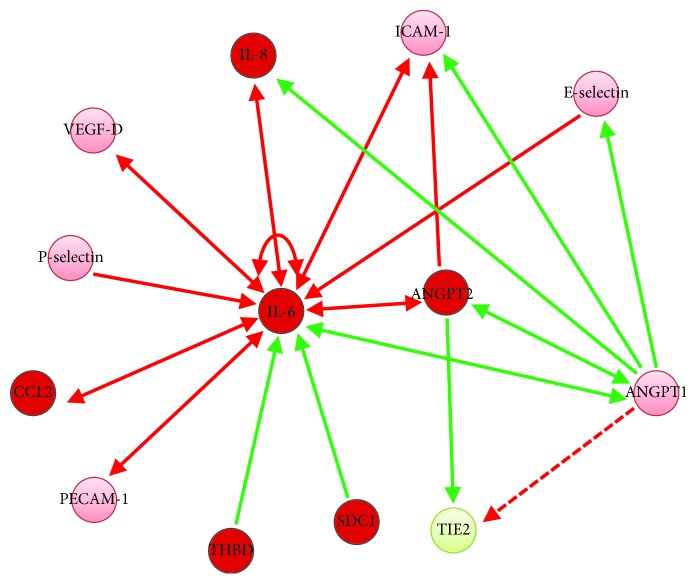
Regulatory network of mediators and markers of vascular inflammation and leakage following CS. The figure shows the interactive association of the analyzed peptides. Proteins being upregulated following cytoreductive surgery (CS) are depicted in red, while those that were downregulated are shown in green. Peptides that are detectable but remained unchanged after 24 hrs are given in light red. Red arrows indicate positive regulation, while green arrows show suppression of expression. The dashed line indicates activation of the TIE2 receptor by its natural ligand ANGPT1.

The interactive network of all analyzed proteins derived from the IPA analysis is depicted in Figure 5. IL-6 influences the activity of other proinflammatory cytokines (IL-8 and CCL2). Furthermore, it exerts a positive feedback on its own regulation and is under the control of THBD and SDC1. The increase in activity of ANGPT2, exerting inhibitory effects both on ANGPT1 and TIE2, decreased serum activity of circulating TIE2. This most likely results in a dysbalance towards leakage-promoting ANGPT2 effects.

## 4. Discussion

With this study, we sought to assess early changes in the serum profile of vasoactive markers and mediators in OC patients during the course of surgery for cytoreduction. Our data suggest that increased activity of IL-6 exerts positive regulation of other proinflammatory mediators and, by upregulating ANGPT2 and suppression of ANGPT1, induces a serum profile that promotes vascular leakage. This may contribute to the observed hemodynamic alterations during CS procedures in OC patients.

### 4.1. Cytokines and Chemokines

During major abdominal surgery, IL-6 was shown to be most early increased in serum compared to other cytokines [12–14]. This might be due to an autocrine feedback loop, positively regulating its own expression [15]. IL-8 and CCL2, both increased after 24 hrs in our patients, are directly positively regulated by IL-6 [15, 16]. In addition, they both exert a positive feedback on IL-6 expression, which could explain the observed strong postoperative increase in these proinflammatory cytokines [17, 18]. IL-8 and CCL2 have been shown to induce endothelial barrier disruption, which is a hallmark of late-stage OC and contributes to hemodynamic destabilization through extensive vascular leakage [19–21]. Furthermore, in murine shock models, IL-8 blockade could ameliorate acute hypotension in response to endotoxin injection, suggesting a mechanistic link to inflammatory vasoplegia [22].

### 4.2. Leakage-Related Mediators

ANGPT1 and ANGPT2 are prominent vascular growth factors with outstanding importance for the regulation of endothelial maintenance and activation. Both are competitive (ant)agonists at the endothelial tyrosine kinase receptor TIE2. Constitutively secreted ANGPT1 mediates endothelial and vascular stabilization through TIE2 phosphorylation and subsequent AKT activation. In contrast, ANGPT2, which is released upon specific trigger signals in case of endothelial activation, blocks ANGPT1 binding to TIE2, thereby destabilizing the endothelial layer and promoting vasodilation by increased iNOS activity [23]. There is a vast body of evidence not only for the symptomatic and prognostic roles but also for the pathogenetic role of a dysbalance between systemic ANGPT1 and ANGPT2 levels in conditions of severe hemodynamic destabilization and shock (such as sepsis, Systemic Inflammatory Response Syndrome (SIRS), and Acute Respiratory Distress Syndrome (ARDS)) [24–26]. As tumor tissue expresses high amounts of angiopoietins during vasculogenesis (mainly ANGPT2), in OC patients, preoperative systemic levels are altered per se and correlate with their oncologic prognosis [27]. During surgical treatment of peritoneal carcinomatosis, ANGPT2 is released into systemic circulation by mechanical trauma of vascular endothelial and tumor cells. We found postoperative ANGPT2 levels that were more than doubled compared to baseline values, which may promote intraoperative IL-6 upregulation [28]. The latter itself, exerting positive feedback, enhances endothelial expression of ANGPT2, thereby further increasing serum levels [29]. IL-6 and ANGPT2 activity were shown to exert an inhibitory influence on ANGPT1 expression, but our data indicate this only in trend. This may shift the ratio between the angiopoietins towards an acute, ANGPT2-driven vascular dysfunction phenotype [29, 30]. This is furthermore aggravated as ANGPT1 itself actually plays a key role in controlling the activity of proinflammatory cytokines such as IL-6 and IL-8 as well as of ANGPT2 [31, 32]. During the course of surgery, the circulating form of the TIE2 receptor was almost reduced to half of the preoperative serum levels. This seems a logical consequence of the observed ANGPT1/ANGPT2 dysbalance, since TIE2 expression is suppressed by ANGPT2 [33]. TIE2 downregulation was found in sepsis and mediates breakdown of endothelial barrier function [34]. Furthermore, ANGPT1-mediated TIE2 phosphorylation was shown to preserve microvascular reactivity, which is lost during major CS for OC, along with hemodynamic deterioration [35, 36]. VEGF isoform D has strong leakage-inducing properties since it binds to VEGF receptor 2 (VEGFR2, Flk-1), thereby contributing to acute hemodynamic destabilization [37]. Since its expression is induced by IL-6 on the one hand, while on the other, VEGF-D itself positively influences IL-6 expression [38, 39], we expected to see elevated serum levels during the course of surgery. Surprisingly, VEGF-D remained unchanged by CS in our patients.

### 4.3. Adhesion Molecules

Serum levels of soluble endothelial adhesion molecules were reported to be acutely increased in critically ill patients, where they correlate with disease severity and prognosis [40]. Thus, we were surprised that neither E- and P-selectin nor ICAM-1 and PECAM-1 were elevated following CS compared to baseline values. A possible explanation could be that they are primarily increased in shock conditions from infectious genesis, while in sterile SIRS, their levels remain rather unaffected [40]. Moreover, in cancer patients, circulating adhesion molecules are increased per se, which may explain the absence of a further elevation during surgery [41]. Expression of selectins as well as CAMs is influenced by IL-6 and *vice versa*, which makes them part of the depicted interactive network [42, 43]. However, as their serum levels do not seem to be altered by CS, they are likely controlled by other regulators (e.g., ANGPT1) [23].

### 4.4. Markers Related to Coagulation

THBD is expressed by endothelial cells. As its name suggests, it converts the mode of action of thrombin from a pro- to anticoagulant molecule by largely increasing thrombin's ability to activate protein C, thereby controlling local blood coagulation. Serum levels of THBD are increased in septic shock [44]. As an integral transmembrane protein located on the surface of endothelial cells, its elevated serum activity is supposed to indicate disturbed microcirculation as well as endothelial damage, which occurs during surgery for cytoreduction in OC [36]. In this respect, it resembles SDC1 [45]. THBD and SDC1 were increased in our patients after 24 hrs compared to the preoperative levels, being indicative of damaged endothelium. Despite an increase of THBD in serum is associated with poorer outcome in sepsis [44], its circulating form as well as that of SDC1 reduces IL-6 expression, thereby serving to control an inflammatory response and preventing overwhelming immune reactions [46, 47].

## 5. Conclusions

Our study is the first to explore acute modifications of interacting mediators of vascular inflammation and dysfunction in OC patients, identifying potential key players for hemodynamic alterations during CS. However, it has some limitations. Future studies should include larger patient cohorts, since restricted *n* number is a limitation of our analysis. Moreover, serum was only sampled prior to and after surgery; thus, no real time course of mediator kinetic can be provided. Furthermore, the number of analyzed peptides is restricted as well, and regulating influence from outside the network which may explain the absence of regulation in our cohort is not covered by our observation. We selected these mediators on the background of their significance for vascular inflammation and leakage. Our results should form the basis for future investigations that will extend that horizon, embedding this small one into broader networks.

## Figures and Tables

**Table 1:** Patient and procedural details.

Parameter	Median (25^th^ and 75^th^ percentiles)
*Patient details*	
*n*	26
Age (years)	70 (57-75)
Body mass index (kg/m^2^)	27.3 (22.5-30.6)
FIGO stage III (*n* (%))	19 (73)
FIGO stage IV (*n* (%))	7 (27)
Neoadjuvant oncostatic chemotherapy (*n* (%))	10 (38)
*Procedural details*	
Duration of surgery (min)	456 (323-542)
Duration of anesthesia (min)	618 (506-689)
Duration of mechanical ventilation (min)	1115 (755-1459)
Fluid intake (ml/kg∗hr)	13.1 (10.6-15.4)
Fluid balance (ml/kg∗hr)	9.4 (7.9-12.6)
Estimated blood loss (ml/hr)	76.1 (41.2-140)
Patients with packed red blood cell (PRBC) transfusion (*n* (%))	15 (58)
Numbers of PRBC units transfused	2 (0-4)
Urine output (ml/kg∗hr)	1.6 (1.1-2.2)
Max. norepinephrine dosage (*μ*g/kg∗min)	0.07 (0.06-0.15)
Max. dobutamine dosage (*μ*g/kg∗min)	0 (0-0.28)
Max. serum lactate intraop. (mmol/l)	1.61 (1.18-1.87)
Hyperthermic intraperitoneal chemotherapy (HIPEC) (*n* (%))	6 (23)
Length of stay in hospital (days)	25 (14-34)
Intrahospital mortality (%)	0
3-month mortality (%)	0

## Data Availability

The data used to support the findings of this study are available from the corresponding author upon request.
